# The Relationship Between Homeownership and the Utilization of Local Public Health Services Among Rural Migrants in China: A Nationwide Cross-Sectional Study

**DOI:** 10.3389/fpubh.2020.589038

**Published:** 2020-12-07

**Authors:** Zicheng Wang, Qiushi Wu, Juan Ming

**Affiliations:** ^1^School of Public Management, Jinan University, Guangzhou, China; ^2^School of Economics and Commerce, Guangdong University of Technology, Guangzhou, China

**Keywords:** homeownership, utilization of local public health services, establishment of health record, attendance of health education, demand-side determinants, supply-side barriers

## Abstract

**Background:** Rural–urban migrants frequently suffer from overrepresented health risks but have poor access to public health services. In China, homeownership status may play a vital role in obtaining local welfare. However, the relationship between homeownership and utilization of public health services has remained largely unexplored. This study aims to address the direct linkage between homeownership and utilization of local public health services among rural migrants in China.

**Methods:** We applied the dataset from the 2017 National Migrants Population Dynamic Monitoring Survey (NMPDMS-2017) to explore the direct relationship between homeownership and the utilization of local public health services. Logit regression was conducted to discuss the associations and to explore the interaction effect.

**Results:** The logit estimations reveal that homeownership is positively related to the establishment of a health record and participation in health education. The interaction term of homeownership and household location and the interaction between homeownership and healthcare center location are related to the increased establishment of a health record. However, the interaction of homeownership and household location merely reveals significant correlations with the health education model.

**Conclusion:** Homeownership is positively associated with the utilization of local public health services among rural migrants in China. Furthermore, homeowners living in urban residential communities and within the vicinity of the healthcare center are more likely to access public health services than those living in other locations.

## Background

China has experienced a massive internal migration since 1978. In 2018, approximately 288 million rural migrants were employed as industrial workers in destination cities. They have become an indispensable part of the urban labor force and an inalienable component of the urbanization of the country (1). However, the *Hukou* system in China divides the population into rural (agricultural *Hukou*) and urban (nonagricultural *Hukou*) parts. Agricultural *Hukou* are assigned to residents who were born in towns and villages; urban *Hukou* are usually for those who were born in cities. The migrants with agricultural *Hukou* can apply for urban *Hukou* when they meet certain criteria, including educational achievement, social insurance, vocational qualification certificate, etc. These criteria are so strict that few can obtain the access. Without a local non-agricultural *Hukou*, rural migrants are mostly excluded from sharing public benefits from the local governments as they have limited access to purchase a house and utilize the public health services. Moreover, their children do not qualify for local public education. Thus, the *Hukou* system still acts as a main constraint preventing rural migrants from migrating to urban cities for employment and reducing their access to local public services and permanent settlement (2, 3). In fact, their temporary status categorizes them as marginalized in destination cities (4, 5). They are often treated as cheap laborers who substitute for urban citizens in undertaking undesirable jobs, but they share no equal rights with the local citizens. Rural–urban migrants are frequently exposed to overrepresented health risks, including communicable diseases (e.g., tuberculosis and sexually transmitted diseases), non-communicable chronic diseases and risk factors, workplace injury and occupational health risks, and poor mental health and women's health (6). However, rural migrants are still regarded as outsiders in terms of access to the local welfare system (7), and urban public health care to them is limited (8, 9).

The serious health risks of rural migrants are among the most concerning issues in the process of new urbanization in China (10). The public healthcare system is almost universally accepted as an important factor that would enhance the health status of migrants (11). In 2009, the central government launched the Essential Public Health Services (EPHS) project to provide basic health services to all urban and rural dwellers (12, 13). This project consists of nine types of basic services, including the establishment of health records, health education, health services for children aged 0–36 months, maternal health services, elder health services, immunizations, infectious disease reporting and treatment, services for patients with type II diabetes, and services for patients with severe mental illness (14–17).

Unfortunately, rural migrants remain less likely to utilize basic public health services compared to local citizens (8, 16). Previous studies have discussed the determinants of public healthcare utilization from the supply and demand sides. Demographic and socioeconomic factors are the major determinants on the demand side (18, 19), while the prevalence of healthcare utilization is differentiated by gender, education attainment, employment status, migration duration, health status, and others (16, 18, 20–22). On the supply side geographical access (distance) is regarded as the main barrier to accessing healthcare services (15).

However, those studies offer no direct discussion on the relationship between homeownership and public healthcare utilization. Homeownership is widely perceived as the crucial milestone in the assimilation process, wherein owning a house represents the long-term commitment to settle down permanently and become a member of the local society (23, 24). Thus, homeownership may establish access to neighborhoods with improved physical and social conditions (25, 26) and, in turn, influence the utilization of local public health services. In the case of China, rural migrants often experience poor living conditions and have a low likelihood of becoming homeowners (27, 28). Homeownership has served as a precondition for attaining membership in major cities and the first step toward obtaining local welfare benefits, such as education, health care, and welfare provision (29, 30).

To address this gap, the current study employs a unique national survey in China to further explore the direct linkage between homeownership and public health services utilization. Three issues are addressed. First, is there a positive association between homeownership and the utilization of local public health services? Second, does any interaction effect exist between homeownership and the location of household/healthcare center access to public health services? Third, does homeownership effect vary in terms of employment status, migration duration, and migration patterns?

This study contributes to the literature in two distinct ways. First, it addresses concurrently the demand- and supply-side barriers to local health service utilization and emphasizes the direct role of homeownership. Second, it is the first study to explore the direct relationship between homeownership and local public health service utilization within China.

## Materials and Methods

### Data Sources

We used data from the 2017 National Migrants Population Dynamic Monitoring Survey (NMPDMS-2017) conducted by the National Health and Family Planning Commission of China. NMPDMS-2017 is an open-access and nationally representative cross-sectional survey of internal migrants aged 15–69 years. These migrants lack the local *Hukou*, and they have lived in local cities for more than 1 month. The survey is conducted in 32 provincial units, which cover all 31 provinces and the Xinjiang Production and Construction Corps (XPCC) of China, 348 cities, and 8,450 communities or villages. The distribution of households surveyed across provincial units ranges from 2,000 to 10,000, including seven sampling units that consist of 2,000, 4,000, 5,000, 6,000, 7,000, 8,000, and 10,000 households. A total of 2,000 households are selected from the least populous unit (i.e., XPCC), and 10,000 households are selected from the largest units, such as Zhejiang and Guangdong provinces.

Sample populations are drawn using the stratified multistage random sampling method with the probability proportional to size (PPS) approach. City units in a province were divided into two tiers. One tier was the mandatory cities, including the provincial capitals and specific major cities. The second tier was all other cities (the city list is available from the https://chinaldrk.org.cn/wjw/#/home). Implicit stratification was implemented in city units according to the population size of the counties and the townships. The primary sampling units were townships, which were drawn from the city units using PPS. The village or neighborhood committee was derived from the townships. The two-phase sampling was applied to draw 20 household samples from targeted village or neighborhood committee. The respondent was the final sampling units, which were obtained using systematic sampling. If more than one individual was sampled in a household, the resample should be carried out until only one qualified migrant was sampled. When it is impossible to access the targeted sample, the NMPDMS team would replace this respondent with another one of the same gender, similar age, and similar residence duration.

Approximately 169,000 households were sampled, according to the definition of migrants. After eliminating missing variable data, we obtained 132,555 valid household samples.

### Variables and Measures

The dependent variable is the utilization of local public health services. This variable is specified as the establishment of a health record and access to health education, which are two popular forms of essential public health services. These two outcomes are binary variables. The former was defined in accord with the question “whether you have establish a local health record”; the latter was derived from the item “which public education you have achieved from the local community in the last year.” In particular, the establishment of health record is coded 1 if the respondent is enrolled in the local health information record system and 0 otherwise. The survey incorporates all five main items of health education, including education in chronic disease prevention, mental health, infectious diseases, reproductive health, and occupational diseases. The dummy of health education attendance is equal to 1 if rural migrants attended at least one of the five items, while rural migrants who failed to attend any of the five items were set to 0.

The focal variable is homeownership derived from the question “What kind of house you actually live in in the urban destination?” Only the respondent had access to answer this question in the survey, which represents the homeownership status of the whole family. Homeownership is coded as 1 if the household owns a commodity house and 0 if they live in self-built housing, social rental, affordable housing, employer-provided dormitories, and other informal housing.

Kiwanuka et al. (31) and Jacobs et al. (32) chose control variables concurrently according to the demand- and supply-side barriers. On the demand side, demographic characteristics (gender, age, and marital status), health status (whether an individual suffers from chronic disease), and the intention of permanent settlement are incorporated^1^. Household location (urban residential community vs. village) and healthcare center location (whether the commuting time to the nearest healthcare center is <15 min) are included as supply-side factors.

### Estimation Strategy

All analyses are conducted using Stata 15.0. Pearson chi-square and ANOVA tests are applied for univariate analysis. The logit regression model is used to explore the direct linkage between homeownership and the utilization of local public health services. The interaction and heterogeneous effects are also discussed through logit regressions.

## Results

### Baseline Characteristics

Table 1 presents the descriptive statistics of the dependent variables and controls. More than a quarter (26.09%) of rural migrants have registered health records, and 60.85% have participated in health education. Rural migrants have a low likelihood of becoming homeowners, given that only 17.32% of them own a house in local cities.

**Table 1:** Univariate analysis.

	**Entire sample**	**Establishment of health records**		**χ^**2**^**	**Health education attendance**		**χ^**2**^**
		**No *N* (%)**	**Yes *N* (%)**		**No *N* (%)**	**Yes *N* (%)**	
**Homeownership**							
No	109,603 (82.68)	82,357 (84.06)	27,246 (78.78)	498.4***	44,655 (86.04)	64,948 (80.53)	670.8***
Yes	22,952 (17.32)	15,613 (15.94)	7,339 (21.22)		7,245 (13.96)	15,707 (19.47)	
**Gender**							
Female	63,639 (48.01)	46,204 (47.16)	17,435 (50.41)	108.2***	23,180 (44.66)	40,459 (50.16)	382.7***
Male	68,916 (51.99)	51,766 (52.84)	17,150 (49.59)		28,720 (55.34)	4,0196 (49.84)	
**Age**^**a**^	36.29 ± 10.78	36.18 ± 10.83	36.63 ± 10.59	24.67***	36.55 ± 11.71	36.13 ± 10.11	1.4e+03***
**Marital status**							
Married	112,455 (84.84)	81,930 (83.63)	30,525 (88.26)	426.5***	41,704 (80.35)	70,751 (87.72)	1.3e+03***
Single	20,100 (15.16)	16,040 (16.37)	4,060 (11.74)		10,196 (19.65)	9,904 (12.28)	
**Education attainment**							
Primary	26,547 (20.03)	20,143 (20.56)	6,404 (18.52)	1,392.0***	11,987 (23.10)	14,560 (18.05)	644.4***
Junior	64,031 (48.31)	47,481 (48.46)	16,550 (47.85)		25,028 (48.22)	39,003 (48.36)	
Senior	27,273(20.57)	19,907(20.32)	7,366(21.30)		9,749(18.78)	17,524(21.73)	
College	14,704(11.09)	10,439(10.66)	4,265(12.33)		5,136(9.90)	9,568(11.86)	
**Health status**							
No chronic disease	125,804 (94.91)	93,148 (95.08)	32,656 (94.42)	22.73***	49,009 (94.43)	76,795 (95.21)	40.21***
Suffered chronic disease	6,751 (5.09)	4,822 (4.92)	1,929 (5.58)		2,891 (5.57)	3,860 (4.79)	
**Permanent settlement intention**							
No	24,686 (18.62)	19,624 (20.03)	5,062 (14.64)	490.8***	11,129 (21.44)	13,557 (16.81)	447.6***
Yes	107,869 (81.38)	78,346 (79.97)	29,523 (85.36)		40,771 (78.56)	67,098 (83.19)	
**Household location**							
Village	39,608 (29.88)	30,883 (31.52)	8,725 (25.23)	483.5***	18,052 (34.78)	21,556 (36.73)	978.2***
Urban residential community	92,947 (70.12)	67,087 (68.48)	25,860 (74.77)		33,848 (65.22)	59,099 (73.27)	
**Healthcare Center (HC) location**							
Distance to the nearest HC more than 15 min	21,984 (16.58)	16,933 (17.28)	5,051 (14.60)	132.7***	9,054 (17.44)	12,930 (16.03)	45.63***
Distance to the nearest HC <15 min	11,0571 (83.42)	81,037 (82.72)	29,534(85.40)		42,846 (82.56)	67,725(83.97)	
*N*	132,555 (100)	97,970 (73.91)	34,585 (26.09)		51,900 (39.15)	80,655 (60.85)	

a *p-values were calculated using ANOVA tests; other p-values were calculated using Pearson chi-square tests*.

*** *Significant (p ≤ 0.01)*.

Of 132,555 respondents, 68,916 (51.99%) are males, 84.84% are married, and the average age is about 36 years old. Approximately one-fifth of rural migrants have no educational attainment beyond primarily school. The majority have completed the 9-year compulsory education. Approximately 5.09% of rural migrants report that they suffered from chronic disease in the previous year, and 81.38% of them intend to settle permanently. With respect to household and healthcare center locations, a mere 29.88% of rural migrants live in the village, and 83.42% spend <15 min commuting to the nearest healthcare center.

### Univariate Analysis

The chi-square and ANOVA tests in Table 1 also indicate that the homeownership is more prevalent among rural migrants with health records than that of their counterparts (21.22 vs. 15.94%). Similarly, the homeownership rate among health education participants is also higher than that among non-participants (19.47 vs. 13.96%). Health record establishment and health education access have fewer male than female attendees (health record establishment: 49.59 vs. 50.41%; health education access: 49.84 vs. 50.16%) and more married than unmarried participants (health record establishment: 88.26 vs. 11.74%; health education access: 87.72 vs. 12.28%). The migrants who can access local public health services are more educated than those who lack such access. In terms of educational attainment, the majority completed senior middle school (health record establishment: 21.30 vs. 20.32%; health education access: 21.73 vs. 18.78%) and college (health record establishment: 12.33 vs. 10.66%; health education access: 11.86 vs. 9.90%). The beneficiaries of public health services typically tend to settle permanently (health record establishment: 85.36 vs. 79.97%; health education access: 83.19 vs. 78.56%). Moreover, the participants in public health services are more likely to live in the urban residential community than in villages (health record establishment: 74.77 vs. 68.48%; health education access: 73.27 vs. 65.22%) and can travel to the nearest healthcare center in <15 min (health record establishment: 85.40 vs. 82.72%; health education access: 83.97 vs. 82.56%).

### Multivariate Logit Analysis

Logit regression was applied to identify the association between homeownership and the utilization of local public health services. Subsequently, odd ratios and 95% CI were calculated.

In the health record model (Table 2), logit estimations reveal that homeownership, gender, age, marital status, educational attainment, health status, permanent settlement intention, household location, and healthcare center location are related to the increasing prevalence of health record establishment. In the health education model, participation in health education is positively associated with homeownership, gender, marital status, educational attainment, permanent settlement intention, household location, and healthcare center location. However, age and health status are related to reduced access to health education.

**Table 2:** Association between Homeownership and Utilization of Local Public Health Services.

	**Health record**			**Health education**		
	**OR**	**CI (95%)**	***p*-value**	**OR**	**CI (95%)**	***p*-value**
Homeownership	1.1926***	(1.1540, 1.2325)	0.0000	1.1883***	(1.1506, 1.2271)	0.0000
Gender (female = 1)	1.1390***	(1.1107, 1.1682)	0.0000	1.2112***	(1.1837, 1.2393)	0.0000
Age	1.0027***	(1.0013, 1.0042)	0.0000	0.9926***	(0.9913, 0.9940)	0.0000
Marital status (married=1)	1.4199***	(1.3623, 1.4798)	0.0000	2.0475***	(1.9760, 2.1217)	0.0000
Junior middle school (ref: primary school or below)	1.1321***	(1.0925, 1.1732)	0.0000	1.2606***	(1.2219, 1.3005)	0.0000
Senior middle school (ref: primary school or below)	1.2085***	(1.1578, 1.2614)	0.0000	1.4693***	(1.4138, 1.5270)	0.0000
College or above (ref: primary school or below)	1.3241***	(1.2579, 1.3938)	0.0000	1.5065***	(1.4372, 1.5792)	0.0000
Health status (Chronic disease = 1)	1.1101***	(1.0484, 1.1754)	0.0000	0.9297***	(0.8822, 0.9798)	0.0060
Permanent settlement intention	1.3617***	(1.3160, 1.4090)	0.0000	1.2203***	(1.1859, 1.2558)	0.0000
Household location (urban residential community = 1)	1.2694***	(1.2330, 1.3068)	0.0000	1.3608***	(1.3271, 1.3953)	0.0000
Healthcare center (HC) location (distance to the nearest HC <15 min = 1)	1.1898***	(1.1494, 1.2315)	0.0000	1.0571***	(1.0257, 1.0895)	0.0000
Constant	0.2169***	(0.2000, 0.2352)	0.0000	1.7368***	(1.6153, 1.8675)	0.0000
Log likelihood		−61716.7			−13403.5	
*N*		132,555			132,555	

*** *Significant (p ≤ 0.01)*.

### Interaction Effect Analysis

The relationship between homeownership and utilization of local public services may depend on the location of the household and the healthcare center. To address this issue, the interaction term of homeownership and household location, and the interaction term of homeownership and healthcare center location are incorporated into the regressions. Table 3 indicates that the interaction term of homeownership and household location and the interaction between homeownership and healthcare center location are related to the increased establishment of health records. However, only the interaction term of homeownership and household location is significantly associated with the increased attendance of health education.

**Table 3:** Interaction effect of geographical accessibility and homeownership.

	**Health records**			**Health education**		
	**Coefficients**	**CI (95%)**	***p*-value**	**Coefficients**	**CI (95%)**	***p*-value**
Homeownership × Urban residential community	1.4141***	(1.2453,1.6057)	0.0000	1.2168***	(1.0734, 1.3794)	0.0020
Homeownership × Distance to the nearest HC <15 min	1.1476***	(1.0491,1.2554)	0.0030	1.0761	(0.9892, 1.1708)	0.0880
Homeownership	1.0358	(0.9520, 1.1271)	0.4140	1.1015	(1.0182, 1.1917)	0.0160
Urban residential community	1.2928***	(1.2549, 1.3319)	0.0000	1.0761***	(0.9892, 1.1708)	0.0000
Distance to the nearest HC <15 min	1.1589***	(1.1157, 1.2038)	0.0000	1.0451***	(1.0115, 1.0798)	0.0080
Constant	0.2227***	(0.2052, 0.2418)	0.0000	1.7575***	(1.6334, 1.8911)	0.0000
Controls		YES			YES	
Log likelihood		−75141.5			−86682.8	
*N*		132,555			132,555	

*** *Significant (p ≤ 0.01); Control variables include gender, age, marital status, education attainment, health status, and permanent settlement intention*.

### Heterogeneous Effect

The utilization of public health services may vary depending on socioeconomic status. To address the different traits, we divide the entire sample into several subgroups in terms of employment status, migration duration, and migration patterns. Regarding employment status, we categorize the sample as employed and unemployed groups. We also propose two migrant groups to determine migration duration, namely, short- (<5 years) and long-term (>5 years) durations. Migration pattern is dichotomized into interprovincial migration and intra-provincial migration.

Logit regression was employed to estimate the heterogeneous effects. The results from Table 4 show that associations between employment status and migration patterns are considerably similar in the health record and health education models. Homeownership is associated with increased local public health service utilization between the employed and unemployed subgroups. Certain positive correlations between homeownership and utilization of local public health services are drawn between interprovincial and intra-provincial groups. In terms of migration duration, significant relationships between long-term and short-term migrants and the prevalence of health record establishment are found. However, a significant association is found only between the short-term group and the health education model.

**Table 4:** Heterogeneous effect across employment status, migration duration, and migration patterns.

	**Health record**					
	**Employment status**		**Migration duration**		**Migration patterns**	
	**Employed**	**Unemployed**	**Duration ≥ 5**	**Duration <5**	**Inter-Provincial**	**Intra-provincial**
Homeownership	0.1737*** (0.0189)	0.1628*** (0.0365)	0.0864*** (0.0216)	0.2457*** (0.0269)	0.2330*** (0.0271)	0.0852*** (0.0215)
Constant	−1.5927*** (0.0480)	−1.4064*** (0.0861)	−1.2466*** (0.0576)	−1.6273*** (0.0625)	−1.5958*** (0.0611)	−1.4216*** (0.0572)
Controls	Yes	Yes	Yes	Yes	Yes	Yes
Log likelihood	−61716.9	−13403.5	−39493.7	−35384.0	−35495.2	−39216.8
*N*	110,009	22,546	65,788	66,767	67,680	64,875
Homeownership^a^	0.1880*** (0.0186)	0.1655*** (0.0362)	−0.0242 (0.0218)	0.2972*** (0.0259)	0.1619*** (0.0254)	0.1139*** (0.0219)
Constant^a^	0.4244*** (0.0421)	0.8187*** (0.0777)	1.2185*** (0.0544)	0.3398*** (0.0518)	0.4063*** (0.0505)	0.7468*** (0.0539)
Controls^a^	Yes	Yes	Yes	Yes	Yes	Yes
Log likelihood^a^	−72052.4	−14562.8	−41048.4	−44609.0	−45353.3	−40787.6
*N*	110,009	22,546	65,788	66,767	67,680	64,875

***Significant (p ≤ 0.01). Control variables include gender, age, marital status, education attainment, health status, permanent settlement intention, household location, and healthcare center location.

a *Represent the estimations from health education model*.

## Discussion

Homeownership is positively associated with the utilization of local public health services among rural migrants. The potential mechanism may be described as follows. First, homeownership represents considerable socioeconomic success in the destination (24), and homeowners may share rising social status (33), thereby enhancing their likelihood of gaining access to local public health services. Second, owning a house in the destinations would encourage social integration among rural migrants (34), who may be encouraged to build greater neighborhood cohesion and social capital compared to renters (35). Homeownership, which symbolizes long-term residential stability, could maintain social ties and decrease stress, which may induce utilization of the essential public health services in the urban destinations (22, 36).

The control variables are also expected to show influence (Table 2). Males are less likely to utilize public health services than females, which is consistent with previous studies suggesting that females are more sensitive to health problems than males (20, 37). Regarding socioeconomic factors, high educational attainment is associated with a high prevalence of health record establishment and health education participation. These findings are consistent with previous evidence that the level of education is associated with the utilization of public health services (21, 38).

The results also reveal that migrants who suffered from chronic disease are more likely to utilize public health services than those who do not. This finding is in line with previous findings that individuals with poor health status are more likely to seek additional health services than those who are healthy (15, 20, 39). Furthermore, permanent settlement intention is related to the increased utilization of public health services, indicating that the expectation of social integration in the urban destination would encourage the seeking of public health services. This conclusion is consistent with Jing et al., demonstrating that social integration is positively related to establishment of the health records among elderly migrants in China (22).

Apart from the demand-side factors, the supply-side factors such as the availability of and convenient access to public healthcare are also regarded as important determinants (32). Migrants who live in the urban residential community and spend a short time traveling to a healthcare center are more likely to establish health records and participate in public health education than those who live in villages and spend a long time traveling to a health center. These findings are consistent with the argument that geographical accessibility is important to local public health service utilization (15, 31).

Homeowners generally reside in neighborhoods with affluent public services. The interaction effect regressions also demonstrate that homeowners living in urban residential communities are more likely to utilize public health services than those living in villages. Similarly, rural migrants who own a house within the vicinity of the healthcare center tend to access public health services. Therefore, homeownership substantially influences those with geographical access to health services, and homeowners in high-quality residential neighborhoods may exhibit high levels of social integration and numerous opportunities to utilize local public health services.

## Conclusion

### Main Findings

Rural migrants remain at a disadvantage for utilizing basic public health services. Homeownership status may play a vital role in attaining local welfare. This study confirms that homeownership is positively related to the utilization of local public health services among rural migrants in China. Moreover, homeowners living in urban residential communities and within the vicinity of the healthcare center are in an advantageous position to access public health services.

### Limitations

This study explores the direct relationship between homeownership and the utilization of local public health services among rural migrants. Our research has two limitations. First, the NMPDMS-2017 reported only limited information about the Essential Public Health Services (EPHS). Therefore, this study discusses only two common forms of EPHS; additional items, including public health services for children and elders, immunizations, infectious disease protection, and services for patients with type II diabetes, should be incorporated in future studies. Second, the study addresses the demand- and supply-side barriers concurrently but only discusses the geographic accessibility due to data limitation. Other factors, such as availability, affordability, and acceptability of local public health services, should be further explored in future studies.

### Implications

Homeownership is positively related to the utilization of public health services. Therefore, policymakers should pay increased attention to the housing attainment and improvement of living conditions of rural migrants. Providing habitable and affordable housing to rural migrants is an urgent issue for the local governments of these urban destinations. Thus, expanding the provision of public rental housing should be considered a major policy direction.

## Data Availability Statement

The data analyzed in this study is subject to the following licenses/restrictions: The NMPDMS are open and available dataset from the Migrant Population Service Center of National Health Commission, the People's Republic of China (PRC). NMPDMS are however available from the Migrant Population Service Center of National Health commission upon reasonable research request. NMPDMS can be requested from the website of Migrant Population Service Center: https://chinaldrk.org.cn/wjw/#/home. Requests to access these datasets should be directed to https://chinaldrk.org.cn/wjw/#/home.

## Author Contributions

ZW took leadership and responsibility for the research activity planning and made substantial contributions to the conception and design of the program. QW worked on the statistical analysis of the data. JM drafted the concept of the paper and participated in finalizing the manuscript. All authors read and approved the final manuscript.

## Conflict of Interest

The authors declare that the research was conducted in the absence of any commercial or financial relationships that could be construed as a potential conflict of interest.

## Footnotes

^1^There are three potential reasons why we cannot incorporate household income into the model. First, according to the analytical framework from Jacobs et al. (32) and Kiwanuka et al. (31), household income is not viewed as the vital factor in accessing health services. Second, previous evidence from housing studies has demonstrated that household income was a significant factor affecting affordability to homeownership, which may result in multicolinearity. Third, the odd ratio of household income is closer to 1, adding it into the estimation, which indicates that income was unimportant for public health services utilization.
